# Changes in children’s attachment security to mother and father after the birth of a sibling: Risk and resilience in the family

**DOI:** 10.1017/S0954579421001310

**Published:** 2021-12-14

**Authors:** Brenda L. Volling, Wonjung Oh, Richard Gonzalez, Lauren R. Bader, Lin Tan, Lauren Rosenberg

**Affiliations:** 1 Department of Psychology, University of Michigan, Ann Arbor, MI, USA,; 2 Department of Human Development and Family Studies, Texas Tech University, Lubbock, TX, USA; 3 Institute for Advanced Study in Toulouse, University of Toulouse Capitole, Toulouse, France

**Keywords:** attachment, baby sibling, children’s behavior problems, coparenting, family systems, fathers, mothers

## Abstract

Changes in children’s attachment security to mother and father were examined for 230 firstborn children (*M* = 31.17 months), their mothers and fathers participating in a longitudinal investigation starting in the last trimester of the mothers’ pregnancy and 1, 4, 8, and 12 months after the birth of an infant sibling. Both parents completed the Attachment Q-set at prenatal, 4, and 12 months. Growth mixture models revealed four latent classes in which children’s attachments were (a) both secure with a modest decline to both parents (68.3%); (b) more secure with father than mother with a steep decline for both (12.6%); (c) both insecure with no change (10%); and (d) more secure with mother than father with a modest increase for both (9.1%). Multi-group latent growth curve analyses revealed that parenting and coparenting differed across families. Children had lower externalizing behavior problems in families with two secure attachments than in families with one secure attachment, either to mother or to father, who, in turn, had fewer problems than children with two insecure attachments. Findings underscore the strengths of a family systems framework to understand attachment relationships with multiple caregivers and the family risks and protective factors that covary with children’s behavioral adjustment after the birth of a sibling.

The development of a secure parent-child attachment is one of the most significant developmental milestones of early childhood (Ainsworth et al., 1978; Bowlby, 1969). The birth of an infant sibling is also a normative developmental transition for many young children with substantial changes in the mother-firstborn relationship, including increases in punitive physical discipline (Baydar et al., 1997), decreases in joint attention and play (Dunn & Kendrick, 1982), a decline in mother-child attachment security (Teti et al., 1996), and instability in the security of the infant-mother attachment relationship (Touris et al., 1995). Disruptions in the mother-child attachment relationship are distressing for young children, often resulting in emotional disturbance and problem behavior (Kobak et al., 2016). Bowlby (1969) actually claimed that strong attachment behaviors could be elicited simply by the “mere sight of mother holding another baby in her arms” (p. 260). Thus, the birth of an infant sibling may be a challenging time for firstborn children as they adjust to changes in the mother-child relationship and witness their cherished attachment figure interacting with another infant.

There are, however, tremendous individual differences in how children react to mothers (and fathers) interacting with their newborn siblings. Volling et al. (2014) found that 1 month after the birth of the sibling, more children approached and joined parent-infant interaction positively than engaged in anxious clingy behavior and avoidance, or made attempts to disrupt parent-infant interaction and dispense with the sibling rival (Volling et al., 2014). Of course, a small percentage (< 3%) of children did protest and interfere in parent-infant interactions and would later develop more externalizing behavior problems in the first year. The current study builds on this research by focusing specifically on changes in children’s attachment security to mothers and fathers after the birth of the infant sibling in an effort to advance a family systems perspective on attachment. A second emphasis was on how changes in children’s attachment security were interrelated with parenting, coparenting, and children’s behavioral adjustment within the family.

## The attachment network of secure and insecure relationships

Recently, Dagan and Sagi-Schwartz (2018) argued persuasively for an integrative framework in understanding how attachment relationships with both mothers and fathers determine children’s behavioral outcomes. They referred to four attachment configurations based on the security and insecurity of children’s attachments in which children’s attachments to parents could be both secure or both insecure, or children could have only one secure attachment to either parent. When predicting children’s behavioral difficulties (e.g., internalizing and externalizing behaviors), findings from studies examining the four attachment configurations find support for an additive hypothesis in which children with two secure attachments have far fewer problem behaviors than those with two insecure and only one secure attachment (e.g., Bureau et al., 2017; Dagan et al., 2021). Others find support for a buffering hypothesis where one secure attachment to either parent offers protection when the attachment to the other is insecure (e.g., Boldt et al., 2014; Bureau et al., 2020; Kochanska & Kim, 2013; Volling et al., 2006).

The current study had longitudinal assessments of firstborn children’s attachment security to both mothers and fathers using the Attachment Q-set across three time points (prenatal before the birth, 4, and 12 months after birth), which allowed an examination of the level of security to both parents before the birth, as well as how children’s attachment security changed once the infant was born. In line with the attachment network framework, the first goal was to use growth mixture modeling (GMM) to identify different attachment configurations, taking into consideration change in children’s attachment security to both their mothers and fathers. GMM is a person-centered, group-based trajectory analysis that relies on latent growth curve (LGC) modeling and allows identification of multiple, unobserved sub-groups (referred to as classes) within a sample that differ with respect to longitudinal change patterns (Ram & Grimm, 2009). We hypothesized that at least four classes of families would be found in line with the four attachment configurations: (1) securely attached to both mother and father; (2) insecurely attached to both; (3) secure to mother, insecure to father; and (4) secure to father, insecure to mother.

Few studies consider children’s attachments to their fathers (Ahnert & Schoppe-Sullivan, 2020; Cowan & Cowan, 2019), and no study has examined changes in children’s attachment security to both fathers and mothers after the birth of a second child. Teti et al. (1996) did find a decline in children’s attachment security to mother after the birth of a second child so we also hypothesized that there may be a decline in attachment security to mother for at least one of the classes. But, with no prior research on children’s attachment to the father, we did not advance firm hypotheses as to how father-child attachment security would change. Stewart (1990) included fathers in his study of 40 families undergoing the transition to siblinghood and found that even though there was change in the mother-child relationship once the sibling was born, similar change in fathers’ interactions with the child was not found. Therefore, there may be different patterns of change for mother-child and father-child attachment security, particularly in families where children may have a secure attachment with one parent, but an insecure attachment with the other.

## Family systems theory and attachment: the parenting and coparenting subsystems

A second goal was to explore the interconnections between parent-child and coparenting relationships in families in which children had secure or insecure attachment relationships with one or both parents. Family systems theory (FST) emphasizes the interdependencies and reciprocal relations among various family subsystems (Cox & Paley, 2003; Volling, 2005). Many of the systemic properties of FST can be brought to the study of children’s attachment relationships with multiple caregivers (Cowan, 1997). For instance, the presence of multiple family members together, as in a mother-father-child triad, creates *emergent family processes* (e.g., coparenting) that are not observable at the level of a parent-child dyad. Importantly, FST also emphasizes the principle of *interrelated wholeness and order* whereby individuals and relationships are part of an integrated system and cannot be understood outside the entire context of interdependent family dynamics. Due to this interdependence, challenges to any one aspect of the family system reverberate throughout all levels of the family (Cox & Paley, 2003). During a normative transition such as the birth of a second child, this means that change in the mother-child attachment relationship will be interrelated with changes in father-child and mother-father relationships. Further, individual family members are both contributing to and being affected by the unfolding of these changing family dynamics over time. This interrelated wholeness of an organized system undergoing change at multiple levels results in different family ecologies in which to understand children’s behavioral adjustment.

A person-centered approach is consistent with one of the basic principles of developmental psychopathology, which is to understand the unique combinations of risk and protective factors that probabilistically determine diverse developmental processes giving rise to diverse psychopathological outcomes (Cicchetti & Rogosch, 1996; Sroufe, 1997). Children’s behavioral adjustment is dependent on the particular combination of risk and protective factors that characterize their family circumstances. Because each resulting class from the GMM may reflect distinct patterns of change across different family subsystems, the risks and protective factors children (and parents) are exposed to within each family may differ. Therefore, some children may experience the transition to sibling-hood with relatively few risks and abundant protection, whereas for others, there is an accumulation of risks and few resources offering protection (Sameroff et al., 1997). Understanding which children will adapt to the transition to siblinghood and which children will experience adjustment problems requires attention to all levels of individual and family functioning.

With these principles in mind, we focused on other changes ongoing in the family system that coincided with changes in children’s attachment security to both mothers and fathers after the birth of an infant sibling. Prior research has documented that children with secure infant-mother attachment relationships have different behavioral outcomes if maternal care changes from sensitive to insensitive at a later point in time (e.g., Belsky & Fearon, 2002). A similar scenario may play out for children after the transition to siblinghood due to changes in the mother-child relationship. Sensitive and responsive parenting is a precursor to the development of secure infant-mother (De Wolff & van IJzendoorn, 1997) and infant-father attachment relationships (although less strongly; Lucassen et al., 2011). Feelings of maternal efficacy and lower parenting stress have predicted attachment security between mothers and preschool children (Teti et al., 1991), as well as across the transition to siblinghood (Teti et al., 1996). Further, parenting stress is negatively associated with maternal sensitivity and, in turn, the security of infant-mother attachment (Booth et al., 2018). Thus, parenting processes were expected to differ across families in which children’s attachment security was high (secure) or low (insecure) with their mother and father. In the current study, we considered parenting stress, parental self-efficacy, parental sensitivity during observations of parent-child interaction, and the use of punitive, parent-centered, discipline when managing children’s misbehavior as indicators of the parent-child subsystem. We hypothesized that declines in attachment security would be associated with increases in parenting stress, the use of more punitive discipline to manage problematic behaviors, and decreases in parental efficacy and parental sensitivity within families.

## Mothers and fathers as attachment figures and coparents

Because children’s attachment security to both their mothers and fathers was a central focus in the current study, we also explored the role of the mother-father subsystem in the form of the coparenting relationship. A recent meta-analysis reported a significant inverse association between interparental conflict, which includes coparenting, and attachment security for children under 5 years of age (Tan et al., 2018). Significant relations have also been found between the quality of the marital relationship and mother-infant attachment security (Frosch et al., 2000; Isabella & Belsky, 1985; Owens & Cox, 1997). Caldera and Lindsey (2006) found significant relations between competitive coparenting and 1-year-old infants’ insecure attachments to both mothers and fathers using the Attachment Q-set. These authors also reported that competitive coparenting predicted less congruence (i.e., similarity) in attachment security across mothers and fathers, suggesting that in families with more coparenting conflict, children’s attachment security to mother and father may differ. This is in line with recent findings by Bureau et al. (2021) who reported more coparenting conflict in families with preschool children when children’s attachment security to mothers was high but low with fathers 3 months earlier. In this case, mothers may be acting as gatekeepers if the father-child attachment is insecure. In all these instances, research indicates the highly interrelated connections between the coparenting relationship and the security of children’s attachment relationships. We hypothesized similar interconnections would be apparent after the transition to siblinghood.

## Children’s adjustment following the birth of a sibling

How firstborn children adjust to the newborn sibling is a common concern expressed by mothers expecting their second child (Affonso et al., 1988). Further, children’s acceptance or rejection of the infant sibling in the early months after the birth predicted the quality of the sibling relationship in the first year (Dunn & Kendrick, 1982; Song & Volling, 2015). Children also exhibited more externalizing problems after the birth if coparenting conflict was high and coparenting support was low before the birth (Kolak & Volling, 2013). Therefore, the final goal of this study was to examine children’s problematic behaviors across the transition and note whether behavioral adjustment differed across attachment configurations. According to Dagan and Sagi-Schwartz (2018), children’s behavioral adjustment depends on the number (0, 1, 2) of secure attachments children have (*additive hypothesis*), with whom children have a secure attachment (mother or father; *hierarchical hypothesis*), and whether a secure attachment to one parent protects children from an insecure attachment to the other (*buffering hypothesis*). A *buffering-horizontal hypothesis* would predict that as long as children had at least one secure attachment to either parent, behavioral outcomes should be similar to those children with two secure attachments. A *buffering-hierarchical hypothesis*, on the other hand, would predict that this protection is afforded only when that security is with one and not the other parent.

Attachment insecurity to mothers may place children at greater risk for behavior problems, regardless of the attachment security to fathers because mothers are often more responsible for child care and spend more time with their children. There are also significant disruptions in the mother-child, but not the father-child, relationship after the birth (Stewart, 1990). Given the purported benefits, however, of fathers’ support after the birth of the sibling (Kreppner, 1988), children may have fewer behavior problems when attachment security to father is high, regardless of children’s attachment security to their mother.

## The current study

The current study was an exploratory, longitudinal investigation examining changes in children’s attachment security to their mothers and fathers in the year following the birth of their infant sibling with three goals: (1) to identify different configurations of attachment security to mothers and fathers by simultaneously modeling the trajectories of mother-child and father-child attachment security over time using GMM; (2) to examine interrelations between children’s attachment security and other family subsystems (parent-child, coparental); and (3) children’s internalizing and externalizing behavior problems in the year following the infant’s birth.

## Method

### Participants

Participants included 241 mothers, fathers, and their firstborn children (*M* = 31.17 months, *SD* = 10.13) participating in a longitudinal investigation examining firstborn children’s adjustment and family dynamics after the birth of a second child. Mothers were 31.6 years (*SD* = 4.22) and fathers 33.2 years (*SD* = 4.78) of age, on average, at the prenatal assessment. Fifty-four percent (*n* = 131) of the firstborns were girls. Families were eligible to participate in the study if mothers were expecting their second child, the biological fathers of the infants were resident, children were between the ages of 1 and 5 years at the time of the infant’s birth, infants were born full-term (>37 weeks of gestation), and both the firstborn and infant were free of physical and developmental delays. The majority of parents were European American (86.3% of fathers, 85.9% of mothers) followed by African American (5.4% mothers, 5.0% fathers), Asian/Asian American (2.9% mothers, 3.7% fathers), and other race/ethnicity (2.1% of mothers and fathers); 3.7 % of mothers and 2.9% of fathers identified as Hispanic. Most parents had at least a bachelor’s degree (79.2% of fathers and 83.9% of mothers), and the median family income was $60,000–$99,999. Mothers and fathers had been married for an average of 5.77 years (*SD* = 2.74). Families were recruited from 2004 to 2008 through advertisements and flyers posted in local obstetric clinics, childbirth classes, pediatricians’ offices, and hospitals. Data collection occurred at five times beginning in the mother’s last trimester of pregnancy (prenatal) and 1, 4, 8, and 12 months following the birth of the infant sibling. A recruitment sample size of 240 was chosen to allow for 15% attrition and a final sample of 200 at 12 months which would allow adequate power (.80) for conducting multilevel modeling with moderate effect sizes (Snijders & Bosker, 2012). The study was approved by the Institutional Review Board of the University of Michigan Medical School (IRBMED).

Of the initial 241 families, 203 families remained at 12 months. Families dropped due to a lack of time, moving from the area, the parents separated, the infant was hospitalized, or they were no longer interested. For families that remained, mothers had higher education, *χ*^2^ (2) = 7.90, *P* < .05, as did fathers, *χ*^2^ (3) = 10.82, *P* < .05, and higher family incomes, *χ*^2^ (3) = 13.94, *P* < .01. The 203 remaining families did not differ from the initial 241 on years of marriage, and mothers’ and fathers’ ages or ethnicity/race. Mplus version 8.2 (Muthén & Muthén, 1998–2017) using maximum likelihood estimation with robust standard errors (MLR) allowed us to retain 230 families for analyses.

### Measures

Data for the current report included assessments of children’s attachment security, the parent-child and coparenting relationships, and children’s problem behaviors. Because some measures were not collected at all 5 time points, we note below in parentheses when each measure was collected. All available data were used in analyses, which meant that the statistical models of change tested in the LGC and GMM analyses were determined by the number of measurement occasions; more complex models of change were applied in instances with 5 times of measurement (see data analysis section for a detailed discussion).

### Attachment Q-set (prenatal, 4, and 12 months)

The Attachment Q-set (AQS Version 3.0, Waters, 1987) was completed by mothers and fathers at the second home visit conducted at the prenatal, 4-, and 12-month time points to assess children’s secure base behavior in the mother-child and father-child relationship. The AQS consists of 90 cards, each of which contains a statement about children’s behavior. Each parent was given the list of 90 behaviors 2 weeks earlier at the first home visit with instructions to observe their children over the intervening 2 weeks. A trained research assistant sat with parents while they sorted the 90 cards into nine piles (10 cards each) ranging from “least characteristic of your child” to “most characteristic of your child.” Mothers and fathers completed sorts separately and based their responses on their own interactions with the child. One parent received the items to sort numbered from 1 to 90 and the other from 90 to 1.

We followed procedures recommended by Teti and McGourty (1996) when administering the parent AQS, including the following: (a) giving parents items beforehand so they could observe their children; (b) keeping parents blind to the fact that the sort was assessing attachment security; and (c) completing the sort in the presence of a trained research assistant to answer questions as needed. Attachment security scores were calculated by correlating mothers’ and fathers’ sorts with a criterion sort representing the hypothetically “most secure” child. Higher correlational scores indicate a stronger association with the criterion sort and a more securely attached child. Mother-father correlations were .312 at prenatal, .290 at 4 months, and .254 at 12 months, all *p*’s < .001. The low magnitude of cross-parent correlations indicates children’s attachment security to mother and father was not highly related, even if significant, so there may very well be families in which children have very different attachment relationships with their mothers and fathers.

In labeling and interpreting the resulting classes from the GMM, we used a convention recommended by Everett Waters (Waters, n.d.), the developer of the AQS, to designate security from insecurity by attending to whether the intercepts for each class (the means at the prenatal visit) were above or below .30. This convention assumes that the proportion of secure (70%) and insecure (30%) attachments using the AQS is the same as that found using the Strange Situation Procedure. For descriptive purposes, the mean security scores at the prenatal time point for the current sample were .43 for mothers and .42 for fathers, and the 30th percentile scores were .36 for mothers and .34 for fathers.

### Parental self-efficacy (P, 1, 4, 8, 12 months)

Both parents completed the *Parental Locus of Control Scale* (PLOC: Campis et al., 1986) to measure parental self-efficacy. The PLOC uses a 5-point Likert scale (1 = *strongly disagree*, 5 = *strongly agree*) to rate parental feelings of confidence in (a) *parental self-efficacy* (10 items; e.g., “what I do has little effect on my older child’s behavior”; *α* = .67–.77; (b) *child control of parents’ life* (7 items; e.g., “my life is chiefly controlled by my older child” ; *α* = .60–.75); and (c) *parental control of child’s life* (10 items; e.g., “my older child’s behavior is sometimes more than I can handle”; *α* = .80–.85). Composite scores were created for each parent at each time by averaging the three subscales. All scores were reverse coded so that high scores reflected a greater sense of parental self-efficacy.

### Parenting stress (P, 1, 4, 8, 12 months)

Mothers and fathers completed 14 items of the *Daily Hassles Scale* (DHS: Crnic & Greenberg, 1990) to assess how hassled and stressed parents felt while completing everyday parenting tasks and managing challenging child behavior (e.g., “child is constantly under foot or in the way,” “child resists or struggles over bedtime”), using a 5-point Likert scale (1 = *no hassle* to 5 = *huge hassle*). Composite scores were created by averaging items for mothers (*α* = .84–.88) and fathers (*α* = .83–.88) separately.

### Sensitive parent-child interaction (P, 4, 8 months)

Both mother-child and father-child interactions were observed during 5 min of free play during home visits at prenatal, 4, and 8 months, and were subsequently coded from video records with rating scales used in the NICHD Study of Early Child Care (NICHD ECCRN, 1999). At the prenatal time point, mothers and fathers interacted separately with the children in dyadic free-play sessions. At both 4 and 8 months, parent-child sessions were conducted while the other parent attended to the infant sibling, and these roles were then reversed. At all 3 times, the order of which parent interacted with the child first was counterbalanced. Researchers brought a bag of toys for the prenatal visit that differed depending on the gender of the child; parents used their own toys at 4 and 8 months. Each 5-min free play session was rated on a 7-point Likert scale (1 = *not at all characteristic*, 7 = *highly characteristic*) for sensitivity/responsiveness (e.g., child-centered interaction tuned into the child’s needs and being sensitive to the child’s agenda). Inter-rater reliability was calculated on a randomly chosen 15% of videos, ICCs = .75–.87 across parents and time.

### Punitive, parent-centered, discipline (4, 8, and 12 months)

The *How Do You Manage Children’s Conflict Scale* (Perozynski & Kramer, 1999) was modified to reflect how parents responded to children’s misbehaviors when interacting with the infant siblings, using a 3-point Likert scale (1 = *almost never*, 2 = *sometimes*, 3 = *usually*). The *parent-centered control* subscale (9 items) was used to reflect punitive, parent-centered discipline (e.g., “told my child that she/he would be punished if she/he did not stop misbehaving” and “used a form of physical punishment to stop my child’s misbehavior”). A composite score was created for mothers and fathers separately by averaging the items at each time (*α* = .69–.77).

### Coparenting conflict and cooperation (P, 4, 8 months)

Both parents completed the 5-item coparenting *cooperation* (e.g., “My spouse says nice things to me about our child”) and 5-item *conflict* (e.g., “My spouse argues with me about our child”) scales from the *Coparenting Questionnaire* (Margolin et al., 2001), using a 5-point Likert scale ranging from 1 = *never* to 5 = *always*. Mothers’ and fathers’ reports of cooperation (*r* = .23–.35, all *p*’s < .01) and conflict (*r* = .49–.53, all *p*’s <.001) were significantly correlated within each time point, so were averaged to create composites reflecting the joint contribution of mothers and fathers to coparenting at the couple level.

### Children’s emotional and behavioral adjustment (P, 1, 4, 8, 12 months)

Mothers and fathers completed the *Child Behavior Checklist* (CBCL/1 ½–5; Achenbach & Rescorla, 2000) at all 5 time points. The CBCL has been used widely to evaluate preschool children’s maladaptive behavior problems. Parents rated 99 items about their children’s behavior on a 3-point scale from 0 = *not true* to 2 = *very true*, which yields two broadband scores for internalizing and externalizing problems. Internal consistency of internalizing problems was high across all time points (*α* = .77–.82) and consistently above .80 for externalizing problems (*α* = .87–.90). Because mothers’ and fathers’ reports were significantly correlated at each time (.26–.50, all *p*s < .001), scores were averaged across parents to create more robust composites that reduced shared method variance, single reporter bias, and the number of variables used in analyses.

### Children’s negative reactivity (P)

At the prenatal time point only, five scales of the *Children’s Behavior Questionnaire* (CBQ: Rothbart et al., 2001) were completed by mothers and fathers to assess children’s temperament using a 7-point Likert scale (1 = *extremely untrue*, 7 = *extremely true*). Only the 13-item *anger/frustration* scale was included in analyses as a control variable because meta-analyses revealed that infant negative reactivity was correlated with parental AQS scores (Cadman et al., 2018). Items were averaged for both mothers, *α* = .77, and fathers, *α* = .73. Because they were significantly correlated, *r* = .533, *p* < .001, mothers’ and fathers’ scores were averaged to create a more robust composite of children’s negative reactivity (Rushton et al., 1983) and to reduce single reporter bias and the number of variables used in analyses.

### Data analysis plan and preliminary analyses

An unconditional latent growth curve model (LGCM) with two parallel growth processes (attachment to mother and father across prenatal, 4, and 12 months) was conducted first to examine overall linear growth patterns with fixed and random effects for the intercepts and linear slopes. The unconditional model was conducted first to determine whether there was sufficient variance in the intercepts and slopes within the sample to proceed with the main GMM analysis looking for classes with different intercepts and slopes (Muthén & Muthén, 1998–2017). Time was centered at the prenatal time point, and paths from the intercept to the observed items were constrained to be 1 for each time point. Paths from the latent linear slope to the observed items were constrained to be 0, 2.5, and 6.5, which corresponded to the unequal spacing between prenatal, 4, and 12 months. This unconditional parallel process model is analogous to a variable-centered analytic approach with random effect terms for the slopes and intercepts.

The growth parameters for the unconditional model can be found in Table 1. The intercepts of attachment security to mothers and to fathers were significantly different from zero, as were the linear slopes. Significant negative slopes revealed overall declines in attachment security to both mothers and fathers over the year. There was significant variance in the intercepts of attachment security to both mothers and fathers reflecting individual variation in attachment security at the prenatal time point. The intercepts of attachment security to mothers and fathers significantly covaried, *cov* = .008, *p* < .001; higher attachment security to mothers was associated with higher attachment security to fathers. There was significant but relatively little variance in the slopes (< .001) for attachment security to both mothers and fathers. The subsequent GMM analyses included fixed effects for both intercepts and slopes that varied across classes, while including the random effects within classes for intercepts only; the variance for slopes within each class was fixed to zero (similar to traditional repeated measures ANOVA). In GMM, the class membership is determined based on the latent variables of the intercepts and slopes in terms of both the fixed and random effects; growth curves of attachment security are similar within a class, but distinct from growth patterns in other classes.

GMM was used to uncover attachment classes (i.e., different family systems) reflecting configurations of children’s attachment security to both mothers and fathers in line with the attachment network proposed by Dagan and Sagi-Schwartz (2018). Residual variances were estimated freely for each time point but were constrained to be equal across classes. Estimated fit indices for 1 (unconditional model) to *k* + 1 class solution models were evaluated to determine which model provided the best fit to the data. Models were not nested, so model comparisons were conducted using a set of fit indices, including the Bayesian Information Criterion (BIC; Schwartz, 1978), the sample size adjusted BIC (SSA BIC; Sclove, 1987), and the Akaike Information Criterion (AIC; Akaike, 1987); lower scores represent better fitting models. The Lo-Mendell-Rubin (LMR) likelihood ratio test (LRT) was also considered for model fit, and entropy, an indicator of the average classification accuracy in assigning individuals to classes, was also examined, with higher scores reflecting better accuracy in classification.

### Multi-group LGCMs

Once the attachment classes were identified, multi-group LGCMs (Duncan et al., 1999) were conducted to determine if trajectories of parenting (e.g., parenting stress, parental sensitivity), coparenting, and children’s behavioral adjustment (i.e., externalizing and internalizing problems) differed across the attachment classes. For these analyses, we relied on theoretical formulations of maladaptive and adaptive change patterns used in prior reports from this research program reflecting three statistical models of change which were determined by the number of time points of available data (see Volling et al., 2017 for details on the modeling contrasts used, the theoretical justification for choosing these three change models, and an example of a similar analytic strategy). Linear growth models reflecting linear change, either a decrease or increase over time, were examined first in all instances. When information was available at 5 times of measurement, two additional models of change were added. The second model added a fixed quadratic effect to the linear change model to assess *sudden and persistent change* where change increased suddenly from prenatal to 1 month after the birth and then persisted over 4, 8, and 12 months. The final model tested for an *adjustment and adaptation response (AAR)* which reflected resilience in the family system and required adding a fixed polynomial contrast over the first 3 time points (prenatal, 1, and 4 months) that tested an increase (or decrease) from prenatal to 1 month that then returned to pre-birth levels by 4 months. As a first step, unconditional LGCMs were conducted separately for each parenting, coparenting, and child behavior variable to determine which of the three change models best fit the data for the overall sample. The best fitting change model for a particular variable was then used in the multi-group LGCMs to compare growth trajectories across the attachment classes. All analyses were conducted with Mplus Version 8.2 (Muthén & Muthén, 1998–2017) using full information maximum likelihood (FIML) estimation.

## Results

### Identifying classes based on changes in attachment security to mother and father

**Figure 1.**
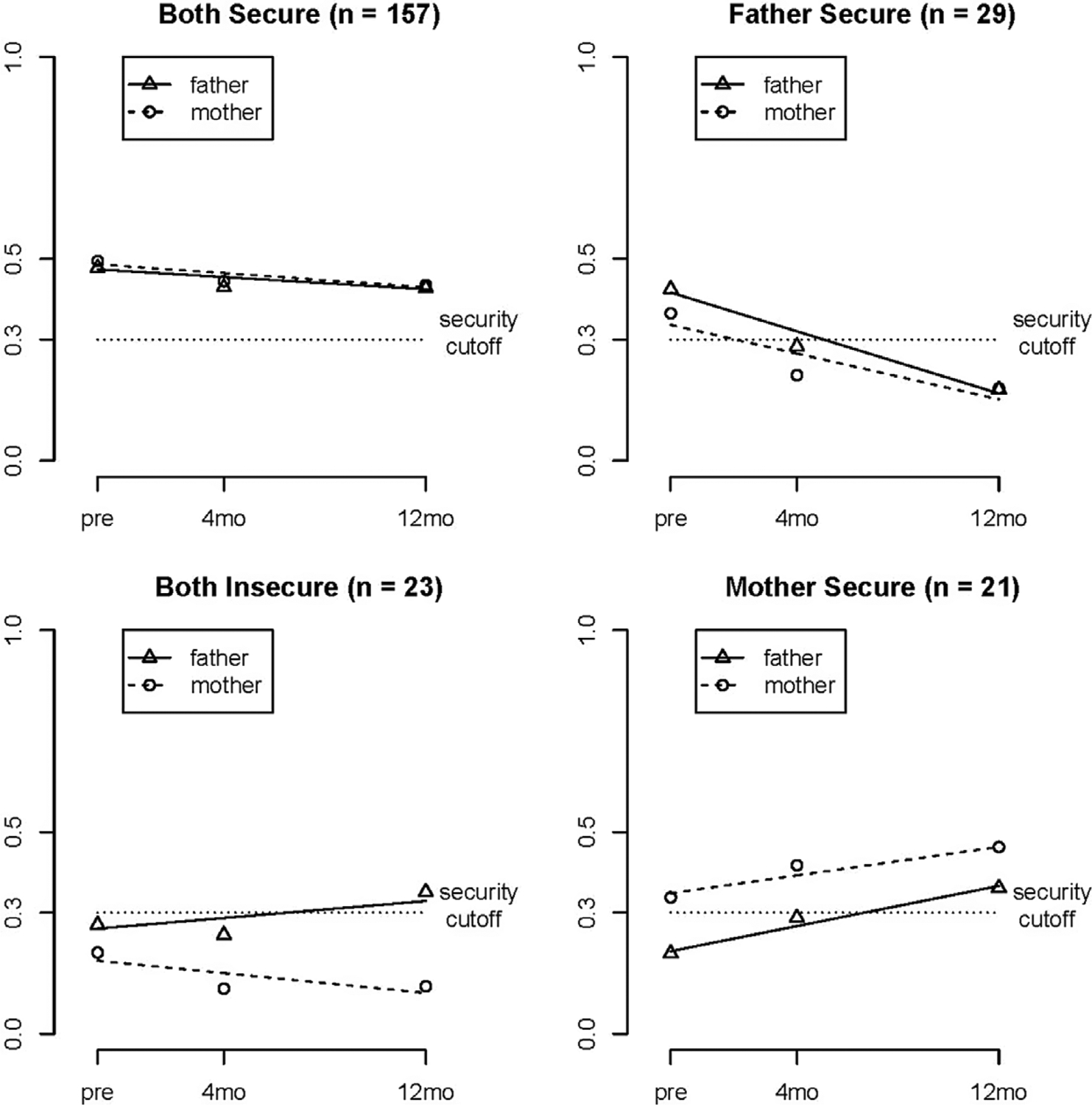
Estimated mean trajectories of GMM 4-class solution for children’s attachment security to their mothers and fathers at prenatal, 4, and 12 months.

To identify classes of families with similar growth trajectories (intercepts and linear slopes), fit indices suggested the four-class model solution was the best fitting model, AIC = −1410.752, BIC = −1314.485, LMR-LRT = .05, over the three-class, AIC = −1400.699, BIC = −1321.623, LMR-LRT = .48, and five-class, AIC = −1417.111, BIC= −1303.654, LMR-LRT = .34, models; the four-class model also had higher entropy (.749) than the five class model (.720). Table 2 presents unstandardized estimates for the fixed effects of the intercepts and linear slopes defining the LGCs of attachment security to mothers and to fathers for each of the four resulting classes. Figure 1 also shows the different trajectory patterns for the four classes with the .30 demarcation used to denote secure from insecure attachments on the y-axis.

Class 1 (C1) constituted the majority of families (*n* = 157, 68.26% of the sample). As seen in Table 2, C1 children had similarly high attachment security to both mothers (.486) and fathers (.474) before the birth of the sibling (intercepts) and significant, but very modest, declines (linear slopes) in attachment security to both parents over the year following the birth. As depicted in Figure 1, the trajectories of attachment security to mothers and fathers were nearly identical. Even though attachment security to both parents declined significantly over time, scores were still above .30 at 12 months, indicating the decline did not reflect a change from secure to insecure. Further, *z*-tests indicated that the intercepts for both mothers, *z* = 13.29*, p* < .001, and fathers, *z* = 13.38, *p* < .001, were significantly higher than .30, and there was no significant difference between mother and father intercepts within C1, *Wald* = .46, *df* = 1, *p* = 50. We referred to this first class (C1) as *children securely attached to both parents with a modest decline in security to both*.

Class 2 (C2) comprised 12.6% of the sample (*n* = 29). Children in this class had higher security scores with fathers (.417) than mothers (.337); yet, this difference was not significant, *Wald* = 1.57, *df* = 1, *p* = .21. Although scores for both parents were above .30 before the birth, only the attachment to fathers was significantly higher (more secure) than .30, *z* = 4.18, *df* = 1, *p* < .001. In addition, the significant negative slopes in Table 2 indicated that attachment security to both parents declined significantly over the year following the birth with scores to both mothers and fathers lower than .30 by the end of the first year (see Figure 1). Attachment security to both parents was lower in C2 before the birth compared to parents in C1 (intercepts in Table 2), but scores also changed from secure to insecure for both parents (less than .30) over time. We referred to C2 as *children more secure to father than to mother with significant declines for both parents from secure to insecure*.

Class 3 (C3) was labeled *children insecure to both parents with no change* (10% of the sample, *n* = 23) because children had low security scores to both fathers (.261) and mothers (.181) before the birth with no significant change in attachment security to either parent over time (see slopes in Table 2). *Z*-tests confirmed that children’s security to mothers was significantly lower than the .30 cut-off, *z* = −2.70, *p* = .007, but children’s security to their fathers was not, *z* = −.83, *p* = .41. Yet, there was no significant difference between the intercepts for children’s security to mothers and fathers within this class, *Wald* = 3.60, *df* = 1, *p* = .06.

The final and smallest class, Class 4 (C4), accounted for only 9.13% of the sample (*n* = 21). The children in this class had significantly higher security scores to mothers (.348) than to fathers (.205), *Wald* = 6.79, *df* = 1, *p* = .009, even though neither was significantly different from .30, mothers, *z* = 1.12, *p* = .26, fathers, *z* = −1.61, *p* = .11. Children’s security to fathers in C4 was significantly lower than children’s security to fathers in C1, *both secure*, *Wald* = 22.44, *df* = 1, *p* < .001, and C2, *father secure*, Wald = 9.64, *df* = 1, *p* = .002, but not C3, the *both insecure* class, *Wald* = .45, *df* = 1, *p* = .50. There were significant increases in children’s attachment security to both mothers and fathers in the year after the birth. C4 was referred to as *children more secure to mother than to father with significant increases in security to both parents*.

The resulting four classes may not map perfectly onto the four attachment configurations based on secure and insecure classifications from the Strange Situation Procedure, most likely because we have information on both the level of security and change in attachment security and are using the continuous scores from the AQS as the measure of attachment security. Yet, the GMM produced four classes resulting in profiles that could be classified based on security to both parents. Therefore, for ease of presentation, we refer to C1 as *both secure*, C2 as *father secure*, C3 as *both insecure*, and C4 as *mother secure* in the remainder of this paper, but remind the reader that classes differed on both intercepts (children’s attachment security to mothers and fathers at the prenatal time point) and slopes (*change* in children’s attachment security).

One-way ANOVAs revealed no significant differences across classes for mothers’ and fathers’ ages or children’s age. Chi-square statistics and Fisher’s exact tests revealed no associations between classes and mothers’ education, children’s gender, and the gender of the infant sibling, but there were significant associations between classes and race/ethnicity of mothers, *Fisher’s* exact = 10.64, *p* = .008 and fathers, *Fisher’s exact* = 22.40, *p* = .007, with a greater percentage of European American mothers in the *father secure* class and a greater percentage of mothers and fathers from other racial/ethnic backgrounds in the *both insecure* class. A significant association between class membership and fathers’ education, *Fisher’s* exact = 22.66, *p* = .004, also revealed that more of these fathers in the *both insecure* class had a high school degree and a lower proportion had a bachelor’s degree. A one-way ANOVA also revealed a significant main effect of class for anger/frustration temperament, *F* (3, 224) = 11.02, *p* < .001; a *post hoc* Tukey test showed that children’s anger/frustration was significantly lower in the *both secure*, *M* = 4.06, *SD* = .61, than in *father secure*, *M* = 4.46, *SD* = .52, *mother secure*, *M* = 4.52, *SD* = .53, and *both insecure*, *M* = 4.68, *SD* = .74, classes. Children’s anger/frustration temperament was controlled in the multi-group LGCMs based on these differences.

### Are there interrelated changes with the parenting and coparenting subsystems?

To address whether parenting and coparenting processes were interrelated with children’s attachment security within each class, we examined the best fitting unconditional LGCMs for each parenting and coparenting variable first. The model fit indices are presented in Table 3 comparing the three change models for variables with 5 times of measurement and are presented in Table S1 in supplemental materials for variables with 3 times of measurement for which we only tested linear growth. The best fitting models are bolded in Table 3 to facilitate the presentation of results that follow. Even though we present findings separately for mothers and fathers to ease presentation, the findings are best interpreted as interrelated changes that are occurring simultaneously across multiple family subsystems rather than independent processes.

### Parenting stress

**Figure 2.**
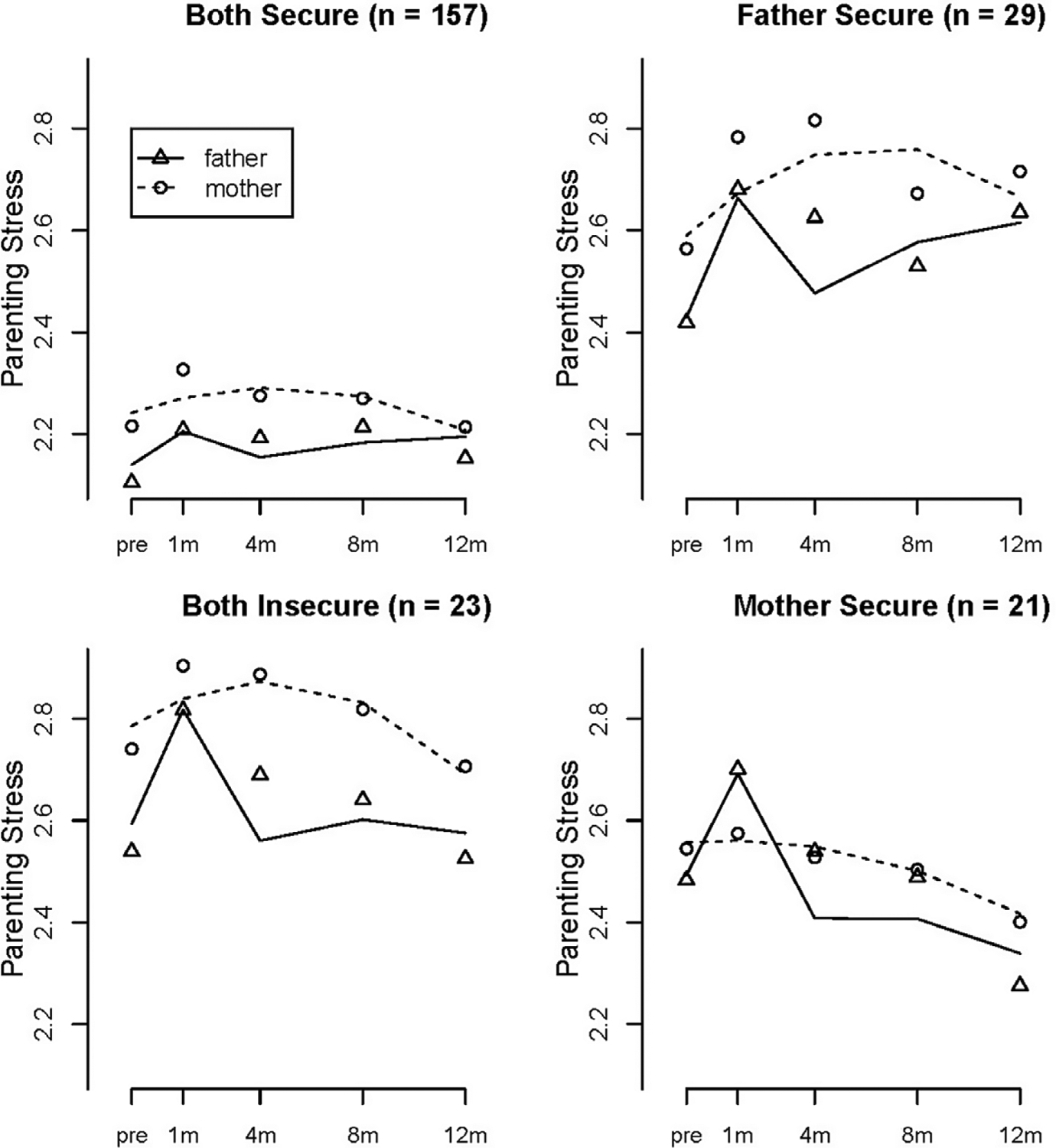
Estimated mean trajectories of maternal and paternal parenting stress at prenatal, 1, 4, 8, and 12 months for each attachment class.

The AAR model was the best fitting unconditional model for paternal parenting stress, and the quadratic model was the best fitting unconditional model for maternal parenting stress (see Table 3). Growth parameters are presented in Table 4. The intercepts indicate that mothers in the *both secure* class reported significantly less parenting stress than mothers in all other classes before the birth, *father secure*, *Wald* = 15.775, *df* = 1, *p* < .001, *both insecure*, *Wald* = 27.970, *df* = 1, *p* < .001 and *mother secure*, *Wald* = 6.642, *df* = 1, *p* = .010. There was a significant linear increase in maternal stress for the *both secure* class, as well as a significant quadratic effect indicating that maternal stress increased gradually over time but then began to decline from 8 to 12 months. Maternal stress also increased linearly over time for mothers in the *father secure* class, but there was no change in maternal stress for the *both insecure* and *mother secure* classes (also see Figure 2).

Table 4 also shows findings for paternal stress. Intercept differences indicated that fathers in the *both secure* class reported significantly less parenting stress before the birth than fathers in *father secure*, *Wald* = 12.719, *df* = 1, *p* < .001 *both insecure*, *Wald* = 25.530, *df* = 1, *p* < .001, and *mother secure*, *Wald* = 12.285, *df* = 1, *p* < .001. There was a significant AAR effect for fathers in every class, with all fathers reporting a sudden increase in parenting stress from prenatal to 1 month after the birth that then decreased by 4 months (see Figure 2). There was a significant linear increase in paternal stress over time in the *father secure* class but a significant decline in paternal stress in the *mother secure* class. The *both secure* fathers reported consistently low parental stress over time and fathers in the *both insecure* class reported consistently high stress over time.

### Parental self-efficacy

The linear model was the best fitting unconditional model for both maternal and paternal self-efficacy (Table 3). Table 4 shows there were significant intercept differences across classes for maternal self-efficacy, with mothers in the *both secure* class reporting significantly more parental efficacy in managing children’s difficult behaviors before the birth than mothers in the *father secure*, *Wald* = 13.226, *df* = 1, *p* < .001, and *both insecure* classes, *Wald* = 35.542, *df* = 1, *p* < .001. Mothers in the *both insecure* class reported less parental efficacy than mothers in the *father secure* class, *Wald* = 6.33, *df* = 1, *p* = .01, and *mother secure* class, *Wald* = 10.26, *df* = 1, *p* =.001. Further, mothers in the *both secure* and *mother secure* classes were the only mothers reporting significant increases (slopes) in parental efficacy over time. See Figure S1 in supplemental materials.

The intercepts for paternal self-efficacy were also significantly different for attachment classes (see Table 4) Here, fathers in *both insecure, Wald* = 21.974, *df* = 1, *p* < .001; *mother secure*, *Wald* = 16.789, *df* = 1, *p* < .001, and *father secure*, *Wald* = 11.836, *df* = 1, *p* = .001, reported lower levels of parental efficacy than fathers in the *both secure* class. Fathers in the *father secure* class also reported a significant decrease in parental efficacy over the year.

### Observations of parental sensitivity

Table 4 reveals findings from the linear models of observed sensitivity for mothers and fathers during home visits at prenatal, 4, and 8 months, which are also shown in Figure S2. Mothers in the *both insecure* class were significantly less sensitive during mother-child interactions than mothers in *both secure*, *Wald* = 4.120, *df* = 1, *p* = .042*, mother secure*, *Wald* = 4.551, *df* = 1, *p* = .033, and *father secure*, *Wald* = 4.490, *df* = 1, *p* = .034. Further, mothers in the *both secure* class increased in their maternal sensitivity over the year, with no changes in maternal sensitivity for any of the other attachment classes. Fathers’ sensitivity did not differ significantly across classes before the birth, but similar to mothers, fathers in the *both secure* class showed a significant increase in their sensitivity to their children throughout the year (see slope in Table 4) but none of the other fathers did.

### Punitive parental discipline

As seen in Table 4, results of the linear models revealed there were no significant differences in mothers’ punitive responses to children starting at 4 months after the infant’s birth (intercepts), but the positive slopes for mothers in *both secure, father secure, and both insecure* classes indicated significant increases in their punitive discipline toward children from 4 to 12 months (also see Figure S3). Fathers in the *both secure* class reported significantly lower levels of punitive discipline starting at 4 months than fathers in *both insecure*, *Wald* = 8.796, *df* = 1, *p* = .003, and *father secure*, *Wald* = 4.083, *df* = 1, *p* = .043, but fathers across all four classes showed a significant increase in their punitive discipline from 4 to 12 months.

### Coparenting

Finally, Table 4 presents the linear results for coparenting cooperation and conflict at the prenatal, 4-, and 8-month time points. With regard to cooperative coparenting, parents in the *both secure* class reported more cooperative coparenting prenatally than parents in the *both insecure* class, *Wald* = 6.952, *df* = 1, *p* = .008, and *mother secure* class, *Wald* = 4.856, *df* = 1, *p* = .028. Parents in the *both secure* and the *father secure* classes did report significant declines in coparenting cooperation throughout the first year following the birth (see Figure S4). As for coparenting conflict, parents in the *father secure*, *Wald* = 4.938, *df* = 1, *p* = .026, and *mother secure* classes, *Wald* = 5.973, *df* = 1, *p* = .015, reported more coparenting conflict than parents in the *both secure* class starting before the birth. Parents in the *both secure* class reported significant increases in coparenting conflict, but even with an increase, their reports of coparenting conflict at 12 months were still lower than reports of coparenting conflict for parents in the other classes (see Figure S5).

### Attachment security and children’s behavioral adjustment

**Figure 3.**
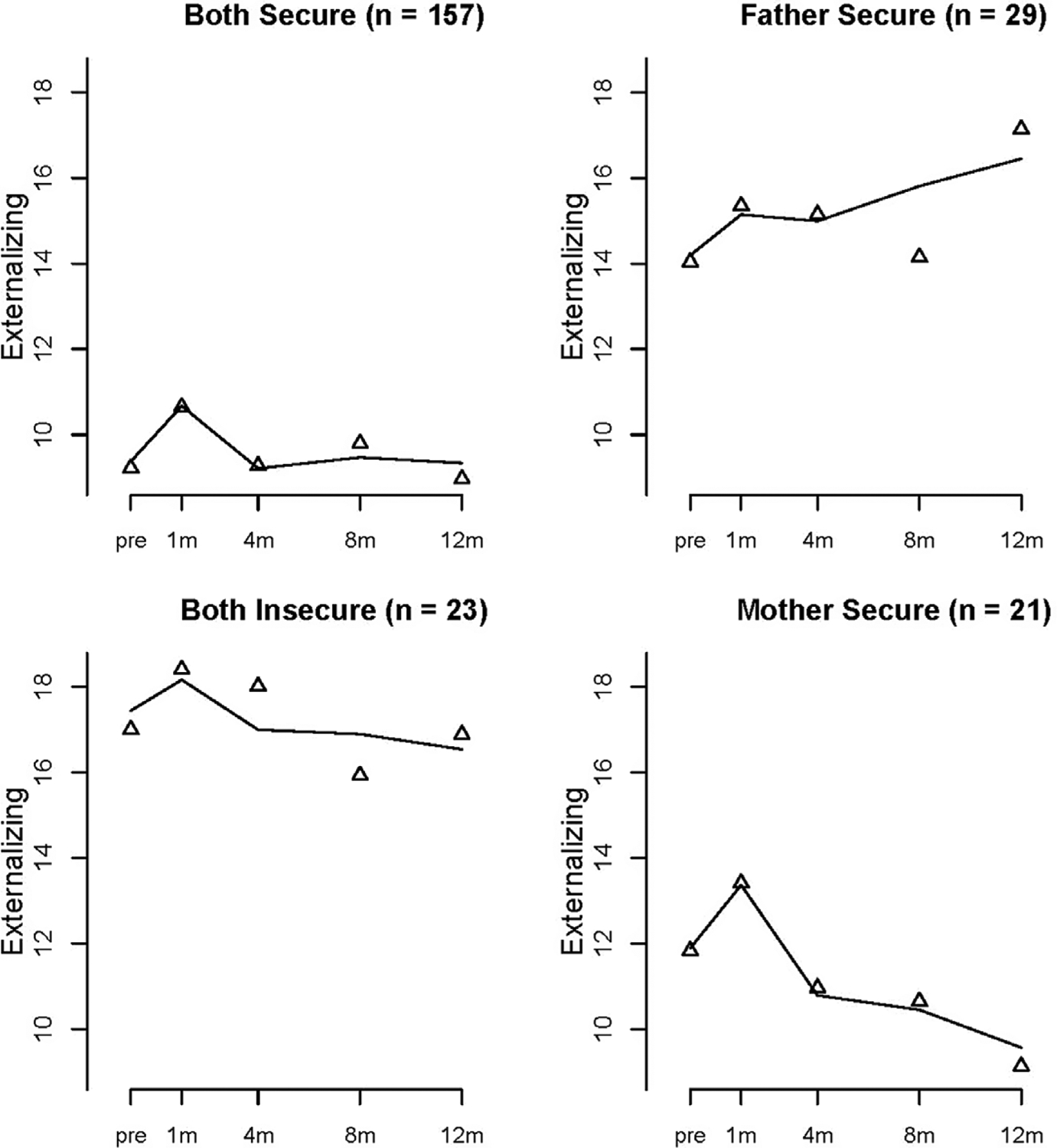
Estimated mean trajectories of children’s externalizing behavior problems at prenatal, 1, 4, 8, and 12 months for each attachment class.

The AAR model was the best fitting model for externalizing problems, and the quadratic model was the best fitting model for internalizing (see Table 3). Figure 3 shows the AAR and the different trajectory patterns of children’s externalizing behavior problems by attachment class. Intercepts in Table 4 show that children in the *both insecure* class displayed the highest levels of externalizing problems prenatally compared to children in *both secure*, *Wald* = 43.601, *df* = 1, *p* <. 001, *father secure*, *Wald* = 5.970, *df* = 1, *p* = .015, and *mother secure*, *Wald* = 12.296, *df* = 1, *p* < .001. Further, children in *father secure*, *Wald* = 33.618, *df* = 1, *p* < .001, and *mother secure*, classes, Wald = 6.986, *df* = 1, *p* = .008, had significantly higher levels of externalizing problems before birth compared to children in the *both secure* class, who had the lowest externalizing scores. Children in *both secure* families did exhibit a significant AAR, with an initial increase from before to 1 month after birth that returned to pre-birth levels by 4 months, indicating these children adapted to changes in the family even if initially stressed immediately following the birth. In contrast, children in the *father secure* class showed a significant increase in their externalizing problems over time that did not subside. Children in the *mother secure* class also showed the initial AAR effect, but then a significant linear decrease in externalizing problems over time (negative slope).

With respect to children’s internalizing problems, growth parameters in Table 4 show intercept differences. Children in the *both insecure, Wald* = 19.922, *df* = 1, *p* < .001, and *father secure* classes, *Wald* = 5.093, *df* = 1, *p* = .024, had significantly higher levels of internalizing problems than children in the *both secure* class before the birth. There were no significant linear slopes or quadratic effects for any of the classes. Table S2 in supplemental materials provides a complete summary of the class differences comparing each class with the *both secure* families.

## Discussion

The primary goal of this investigation was to examine changes in the security of mother-child and father-child attachment relationships in the year following the birth of an infant sibling. The current study advances research on the transition to siblinghood and the study of attachment in several key areas. First, we included information on children’s attachment security to both their mothers and fathers across three longitudinal time points starting from before to a year after the birth of the infant sibling. Second, trajectories of children’s attachment security to both parents were modeled jointly, rather than separately, to identify different classes or attachment configurations before the infant’s birth and how children’s attachment relationships might change across the year following the birth. Finally, we examined other interrelated changes in parenting, coparenting, and children’s behavioral adjustment within each attachment class. In the end, we were able to demonstrate that exposure to different risks and protective factors within each family system over time can explain why some children develop more behavior problems than others across the transition.

### Family systems and attachment security to mothers and fathers

Prior research reported significant declines and instability in children’s attachment security to mothers across this transition (Teti et al., 1996; Touris et al., 1995), yet no study had considered the security of the father-child attachment relationship, even though it is theorized that fathers play a supportive role for children during this stressful transition. Because father-child interaction changed little in the early months after the birth compared to the dramatic changes in mother-child interaction (Stewart, 1990), knowing how the security of father-child attachment might compensate for disruption in the mother-child attachment is worthy of investigation.

The GMM analyses revealed four attachment classes that mapped closely onto the four attachment configurations, showing that children varied in their attachment security to mothers and fathers before the birth and throughout the year after the birth. Given that more children have secure than insecure attachments to their parents, it was not surprising that the largest attachment class consisted of *both secure* families (68.3%) in which children’s attachment security to both mothers and fathers was high before the birth, even if there was a very modest decline in attachment security to both parents over time. For the second *father secure* class (12.6%), children’s attachment security to fathers was higher than to mothers before the infant was born, but unlike the modest decline found in the *both secure* families, there was a significant and dramatic decrease in attachment security to both parents, going from secure to insecure over the year. Children’s attachment security to both parents was low in the third class (10%), labeled *both insecure*, and remained low across time with no change. Finally, the smallest class (9.1%) was labeled *mother secure* because children’s attachment security to mothers was significantly higher than with their fathers, but there was a significant increase in attachment security to both parents across the year following the birth. These findings revealed clear support for different family configurations based on change in children’s attachment security to both their mothers and fathers.

These findings are noteworthy for another reason. Recall that findings from the initial unconditional model actually revealed an overall decline in children’s attachment security to both mothers and fathers for the sample as a whole. But, the key insight here from GMM over a variable-centered approach is that one can uncover unobservable classes in which change in attachment security does not align with this overall pattern. Indeed, we found evidence that children’s attachment security to fathers and mothers declined (father secure), increased (mother secure), and evinced no change (both insecure) over time depending on the class.

### Attachment security is part of a changing family dynamic

Not only were these changes in children’s attachment security to mothers and fathers interrelated but they also coincided with increases and decreases in other aspects of the family system and children’s adjustment. Finding that changes in parenting and coparenting were also interrelated in predictable ways with changes in mother-child and father-child attachment relationships reflects the wholeness of the family and spillover across family subsystems. Understanding the paths linking parenting to child functioning, or coparenting to parenting, can only be understood within the nexus of family experience as a whole (Davies et al., 2004). Because we strongly advocate for continuing to investigate the interdependent nature of family relationships when studying children’s attachments to multiple caregivers, we discuss the interconnections among family subsystems and children’s behavioral adjustment for each attachment class in the remainder of this paper. Our goal is to demonstrate the different interrelated family dynamics unfolding within each family system over the transition and, in turn, how children and their parents were exposed to different risks and protective factors that either promoted resilience during a stressful transition or set the stage for a family in crisis.

### Family system 1 (both secure)

Not only was children’s attachment security to both mothers and fathers high in the *both secure* families, but parenting, coparenting, and children’s behavioral adjustment differed in comparison to other families, and particularly compared to families in which children’s attachment security was judged to be *both insecure*. Consistent with an additive hypothesis, we did find that children functioned better in the *both secure* than the *both insecure* families, with less externalizing and internalizing behavior problems both before and after the birth of the infant sibling. Further, being securely attached to both parents afforded added protection, as children in the *both secure* families had less externalizing behaviors than children secure to only one parent who, in turn, had less externalizing behaviors than children in the *both insecure* families.

Together, mothers and fathers in *both secure* families also reported significantly less parenting stress than parents in any other families and both parents reported a greater sense of parental efficacy in managing their children’s difficult behaviors than parents in the *both insecure* families; this was already the case before the birth of the infant. Further, both mothers *and* fathers in these families were the only parents to increase in parental sensitivity during parent-child interactions over the year following the birth and used less punitive discipline starting at 4 months to manage their young children’s misbehaviors when they antagonized the infant. Although these parents increased slightly in punitive discipline from 4 to 12 months, so did many of the parents in the other families, which may be a response to normative changes as infants mature and sibling squabbles emerge (Dunn & Kendrick, 1982; Volling et al., 2017). As couples, they reported significantly more coparenting cooperation than other families prenatally and over the year. Even though coparenting cooperation declined and coparenting conflict increased after the birth, these couples were still more cooperative coparents and engaged in less coparenting conflict at the end of the first year than couples in the other three family configurations. All in all, children in the *both secure* families lived in a caregiving environment that offered protection and promoted resilience across a stressful family transition. The *both secure* families also constituted the largest class, which stands in contrast to portrayals of the transition as a time of problematic disruption in the mother-child relationship (e.g., increased punitive discipline, confrontations between mothers and children). Again, one of the advantages of a person-centered approach is the ability to uncover distinct classes in which parents and children fare well from those in which families are at higher risk for difficulties.

Even though most children lived in families in which both their attachments were secure, the transition was not completely stress-free for these parents and children. Indeed, both mothers and fathers reported an increase in parenting stress immediately following the birth that coincided with an increase in children’s externalizing behaviors. By 4 months, paternal stress and children’s externalizing problems had declined to pre-birth levels (the AAR effect), with maternal stress declining more gradually over the year. We can only speculate as to why maternal and paternal stress changed differently in these families. Even though fathers have increased their child care involvement over the past decades and male-breadwinner gender norms have waned, mothers still assume most of the child care responsibilities (Hofferth & Lee, 2015). Further, many men still see providing for their families as central to their identity as a father and what it means to be a responsible father (Christiansen & Palkovitz, 2001; Dechant & Rinklake, 2016), which may explain why Stewart (1990) found that many fathers in his study often increased their work hours before the birth of a second child in anticipation of the impending financial costs associated with raising two children. Moreover, many men in our sample had returned to work by 4 months (Kuo et al., 2018). Because of these greater child care demands on mothers following the birth and fathers returning to work within months of the birth, parenting stress may decline more gradually over the year for mothers compared to fathers.

### Family system 2 (both insecure)

Children and parents in the *both insecure* families fared poorly across all levels of child and family functioning. These children had the highest externalizing problems compared to children in any of the other families, even before the infant was born, and their externalizing behaviors remained high over time. They also had more internalizing problems than children in the *both secure* families. Mothers reported the lowest scores on parental efficacy when managing their children’s difficult behavior and were also the least sensitive in their interactions with children before the birth of their infant than any other mothers. Fathers, too, appeared to have difficulties in their parenting roles, as they reported significantly more parenting stress, felt less efficacious in managing difficult child behavior, and used significantly more punitive discipline in response to misbehavior starting at 4 months compared to *both secure* families. Both parents were also less cooperative coparents which may reflect the challenges of mothers and fathers working conjointly to manage the care and discipline of their children both before and after the birth. Additionally, these challenging dynamics surrounding family life persisted over the year with little evidence of abating. In sum, there were a greater number of risks, and fewer protections, in the family environments surrounding these children that may have accounted for the dual-insecurity of the mother-child and father-child relationships, as well as the high levels of children’s problem behaviors.

### Family system 3 (father secure)

Even though attachment security to fathers was higher in these families than for mothers, fathers still reported challenges in their parenting role. Compared to fathers in *both secure* families, they reported feeling less efficacious in handling their children’s difficult behaviors and more parenting stress before the infant was born, and starting at 4 months, used more punitive discipline when children antagonized the infant. These couples also reported more coparenting conflict and both parents increased in their use of punitive discipline across the year as children’s externalizing behaviors increased. Children in the *father secure* families were the only children to show a dramatic increase in externalizing behavior problems over time and already had higher externalizing (and internalizing) behavior problems before the sibling was born compared to *both secure* families. In prior analyses, Volling et al. (2017) found that most firstborn children, on average, increased in externalizing problems in the month after the birth, but by 4 months, had returned to their pre-birth levels, the AAR effect. Rarely, did we see a pattern reflecting a developmental crisis (i.e., a continual increase in externalizing behaviors in the following year). But, this is exactly what occurred for children in the *father secure* families. When attachment security to mother was low, regardless of the higher attachment security to fathers, parents and children struggled through the transition and the year following the birth.

The fact that a more secure father-child attachment did not appear to buffer children from the effects of having a less secure mother-child attachment was one of the most surprising findings from this research, particularly given that prior studies have reported that children with only one secure attachment (to either mother or father) were often no different on behavioral outcomes such as externalizing behaviors than children with two secure attachments (e.g., Kochanska & Kim, 2013). Kochanska et al. (2009) have argued that children’s history of attachment security or insecurity serves to moderate future parent-child dynamics, with different underlying processes responsible for the emergence of problem behaviors. A similar explanation may be at play here with different family dynamics unfolding over the course of the transition for each of the families. Understanding how mothers and fathers work together to manage this transition and become the parents of two children may be critical in interpreting our findings. As such, the links between children’s functioning, parenting, and coparenting can only be understood in the context of collective family experiences and family processes unfolding over time.

As the birth approached for the *father secure* families, mothers were already reporting feeling ineffective in handling their children’s troubling externalizing behaviors. Once the infant was born and mothers assumed the primary responsibility for infant care, this loss of maternal attention and further disruption in an already insecure mother-child attachment relationship may have contributed to further declines in children’s attachment security to their mothers and increases in problematic behavior. Fathers often step in and are more involved with the care of the firstborn shortly after birth (Kuo et al., 2018), but fathers in these families also reported feeling ineffective in managing their children’s difficult behaviors, and there was an increase in parenting stress and coparenting conflict over the following months. Negativity and conflict in the coparental and marital subsystems can spillover and adversely affect the parent-child relationship (Erel & Burman, 1995), which may explain the increase in punitive discipline by both parents in response to children’s escalating externalizing behavior and the precipitous decline in children’s attachment security to both parents over the year. We suspect it is this confluence of cascading interrelated family processes that set the stage for a family in crisis.

### Family system 4 (mother secure)

Similar to mothers in the *both secure* families, mothers in the *mother secure* families were confident in their parenting abilities to manage their children’s difficult behaviors even before the birth and increased in their confidence over time. This confidence may be one reason why these mothers did not report increased parenting stress across the transition or increased in their use of punitive discipline to manage children’s misbehaviors directed at the infant later in the year (although fathers did). This increase in children’s attachment security to mothers after the birth was rather unexpected because improvement in mother-child relationships after the birth of a second child is rarely, if ever, discussed. Coinciding with the increase in children’s attachment security to mothers was a steady decline in children’s externalizing behaviors. Prior studies have reported that even in the midst of a chaotic home environment, a mutually responsive and positively oriented mother-child relationship in the preschool years protected children from developing problem behaviors (Goffin et al., 2018; Supplee et al., 2007; Wilhoit et al., 2021). Such a positive relationship orientation may also assist children across the transition to siblinghood. But, children’s attachment security to their fathers also increased over time in these families, even if low before the birth. Fathers did report less parenting efficacy and more parenting stress in managing difficult child behaviors before the birth compared to *both secure* families, and this may be one reason why children’s attachment security to fathers was low in the first place. Both parents reported less cooperative coparenting and more coparenting conflict than *both secure* families, so mothers early on may have been acting as gatekeepers and closing the gate when fathers were ineffective in managing children’s behaviors (Bureau et al., 2021; Caldera & Lindsey, 2006; Schoppe-Sullivan et al., 2015), but may have been more willing to open the gate over time. One of the strongest predictors of paternal engagement with young children is the quality of the mother-child relationship (Cowan et al., 2009). In this regard, Baker et al. (2018) found that when fathers viewed mothers as more affectionate, encouraging, and willing to compromise as coparents, they were also more engaged in playful and cognitively stimulating interaction with their young children. Perhaps similar positive spillover across the mother-child and father-child relationships transpired in the *mother secure* families.

In sum, different family dynamics played out over the course of the transition and the following year for children and their parents in each of the classes, which were no doubt responsible for differences in children’s behavioral outcomes after the birth of their infant sibling. The behavioral outcomes for children in the *mother secure* and *father secure* families differed, with one group experiencing increases in children’s security to both parents and a decrease in problem behaviors and the other, decreases in security to both parents and increases in problem behavior. Interestingly, neither the *mother secure* nor *father secure* families differed before the birth of the infant sibling on any child, parenting, or coparenting variables (see Table S3 summary in supplemental materials). What appeared to account for the differing adjustment outcomes was the unfolding of different family dynamics over the year following the birth.

### Limitations and future directions

Despite the family perspective offered here and the many strengths of the longitudinal research design and group-based trajectory analyses, we must also acknowledge the limitations of this research. First, all families were two parent, mother-father families in the Midwestern U.S. from predominantly middle class, mostly college-educated, European American families. Additional research is needed to examine how the transition is managed in families from diverse racial/ethnic backgrounds, in families with same-sex parents, and in families with limited financial resources. Second, the validity of the parent AQS in comparison to the observer AQS has been questioned by some (Cadman et al., 2018). Although the use of observer AQS sorts may have yielded different results from those reported here with the parent AQS, the longitudinal design necessitated a methodology that allowed repeated measurements with two parents while reducing the number of home visits and data collection burden on families. Understanding secure base behavior from the parents’ perspectives may also be worthy of further investigation, and perhaps future research would benefit by analyzing both observer and parent AQS scores together in an analogous manner to the GMM used here. Third, the multi-group latent growth models involved multiple models being tested using multiple parent reports, which may have increased the rate of Type I errors and chance findings. Fourth, there are other changes in family functioning besides parenting and coparenting that may account for some of the current findings, including changes in perinatal depression and partner relationship quality (Volling et al., 2015; Volling et al., 2019), which were beyond the scope of the current paper. Finally, there is another child in these families, the infant siblings, who are also intertwined within these family subsystems and forming their own attachments to mothers and fathers across the first year. Our focus here was on the firstborn children, but future research on children’s attachment relationships may want to move beyond a focus on a single parent-child dyad and be mindful of the many different attachment figures in the lives of young children and the many different children in the lives of parents. The current study was the first to consider children’s attachment security to both their fathers and mothers after the birth of an infant sibling, a developmental transition that affects large numbers of young children and their parents. We invite others to replicate and extend these findings to other family circumstances so that in the end, we can assist all children undergoing the transition to siblinghood.

## Supplementary Material

1

## Figures and Tables

**Table 1.** Unconditional latent growth curve model for parallel growth parameters of children’s attachment security to their mothers and fathers (*N* = 230)

	Attachment security to father				Attachment security to father			
	Intercept		Linear slope		Intercept		Linear slope	
Mean (*SE*)	.418***	(.011)	−.009***	(.002)	.412***	(.010)	−.007**	(.002)
Variance (*SE*)	.021***	(.002)	0.00	-	.017***	(.002)	0.00	-

*Note*. The random effect of the slope of attachment for mothers and fathers was set to zero in the unconditional latent growth curve model due to limited variance.

** *p* < .01.

*** *p* < .001.

**Table 2.** Unstandardized growth parameter estimates of classes based on the linear trajectory of children’s attachment security to their mothers and fathers using growth mixture modeling *(N* = *230)*

				Trajectory classes				
	Class 1 Both secure *n* = 157 (68.26%)		Class 2 Father secure *n* = 29 (12.60%)		Class 3 Both insecure *n* = 23 (10%)		Class 4 Mother secure *n* = 21 (9.13%)	
Growth parameters	Mother	Father	Mother	Father	Mother	Father	Mother	Father
Intercept	.486***^a^	.474***^(a)^	.337***^b^	.417**^(a)^	.181***^c^	.261***^(b)^	.348***^b^	.205**^(b)^
Linear slope	−.009**	−.008***	−.028***	−.038***	−.012	.011	.018***	.025**

*Note*. Both secure = Children’s attachment security to both mother and father high with security decreasing for both over time; Father secure = children’s attachment security to father higher than security to mother with both decreasing over time; Both insecure = children’s attachment security to both mother and father low with no change over time; Mother secure = children’s attachment security to mother higher than security to father with both increasing over time. Intercepts with different superscripts across classes for mothers (and in parentheses for fathers) are significantly different at *p* < .05.

** *p* < .01.

*** *p* < .001.

**Table 3.** Unconditional model comparisons of linear, quadratic, and adjustment and adaptation change latent growth models for children’s problem behavior, parental stress, and parental self-efficacy measured at 5 times (prenatal, 1, 4, 8, and 12 months)

Model fit index	Externalizing problems	Internalizing problems	Maternal stress	Paternal stress	Maternal efficacy	Paternal efficacy
Linear model						
χ2 (10)	39.492	29.105	46.413	70.533	**5.118**	**13.405**
p	.000	.001	.000	.000	**.883**	**.202**
CFI	.965	.974	.953	.926	**1.000**	**.994**
TLI	.965	.974	.953	.926	**1.007**	**.994**
RMSEA	.113	.091	.123	.158	**0**	**.039**
AAR model						
χ^2^ (6)	**10.654**	17.006	22.634	**26.978**	1.564	5.267
p	**.100**	.009	.001	**.000**	.955	.510
CFI	**.995**	.985	.979	**.974**	1.000	1.000
TLI	**.991**	.975	.965	**.957**	1.011	1.002
RMSEA	**.058**	.089	.107	**.120**	0	0
AIC	**5870.074**	5237.285	1090.832	**1006.753**	392.183	346.431
Quadratic model						
χ^2^ (6)	32.524	**14.588**	**19.630**	31.952	4.048	9.904
p	.000	**.024**	**.003**	.000	.670	.129
CFI	.969	**.989**	**.983**	.968	1.000	.993
TLI	.948	**.981**	**.971**	.947	1.005	.988
RMSEA	.139	**.079**	**.097**	.134	0	.054
AIC	5891.845	**5234.867**	**1087.828**	1011.728	394.666	351.068
Model fit diff: Δχ^2^ (4)						
Linear vs. AAR	28.838***	12.099*	23.779***	43.555***	3.554	8.138
Linear vs. Quadratic	6.968	14.517**	26.783***	38.581***	1.070	3.501

*Note*. AAR = Adjustment and Adaptation Response included a polynomial contrast to test an increase (or decrease) from prenatal to 1 month (adjustment) and a subsequent decrease (or increase) from 1 month to 4 months (adaptation).The quadratic effect was used to test a developmental or family crisis model reflecting a sudden (increase from prenatal to 1 month) and persistent change that remained high across the year (4, 8, and 12 months). Variables in the table were measured at all five measurement occasions (prenatal, 1, 4, 8, and 12 months) allowing a test of linear, quadratic and AAR change trajectories. The best fitting model of the three change patterns is noted in bold for each measure and these models were used in the multi-group latent growth curve analyses. See Volling et al. (2017) for a complete description of the theoretical rationale for testing these change models and the model details for conducting these analyses.

* *p* < .05.

** *p* < .01.

*** *p* < .001.

**Table 4.** Unstandardized growth parameter estimates of children’s behavior problem, parenting, and coparenting (controlling for children’s angry/frustration temperament) for each attachment class

Growth parameters	Both secure *n* = 157	Father secure *n* = 29	Both insecure *n* = 23	Mother secure *n* = 21
Externalizing problems				
Intercept	9.766***^a^	14.368***^b^	17.697***^c^	12.438***^b^
Linear slope	−0.066	0.320*	−0.179	−0.442***
AAR	0.388***	0.181	0.257	0.546*
Internalizing problems				
Intercept	5.907***^a^	7.639***^b^	9.598***^b^	7.482***
Linear slope	−0.017	0.277	−0.091	−0.423
Quadratic slope	0.001	−0.012	0.041	0.031
Maternal parenting stress				
Intercept	2.242***^a^	2.589***^b^	2.786***^b^	2.557***^b^
Linear slope	0.035*	0.096*	0.066	−0.008
Quadratic slope	−0.006*	−0.013^†^	−0.012	−0.005
Paternal parenting stress				
Intercept	2.157***^a^	2.490***^b^	2.661***^b^	2.559***^b^
Linear slope	0.006	0.019*	−0.013	−0.034*
AAR	0.017*	0.062**	0.068**	0.067**
Maternal parenting efficacy				
Intercept	3.920***^a^	3.679***^b^	3.439***^c^	3.771***^ab^
Linear slope	0.011**	−0.008	0.002	0.031***
Paternal parenting efficacy				
Intercept	3.980***^a^	3.749***^b^	3.622***^b^	3.645***^b^
Linear slope	0.002	−0.021**	0.018	0.015
Maternal sensitivity				
Intercept	4.671***^a^	4.769***^a^	4.142***^b^	4.820***^a^
Linear slope	0.053**	0.024	0.061	0.020
Paternal sensitivity				
Intercept	4.475***	4.261***	4.210***	4.416***
Linear slope	0.052*	0.061	0.087	0.024
Maternal punitive discipline				
Intercept	1.631***	1.748***	1.790***	1.577***
Linear slope	0.098***	0.123***	0.104*	0.053
Paternal punitive discipline				
Intercept	1.501***^a^	1.647***^b^	1.711***^b^	1.575***
Linear slope	0.100***	0.121***	0.092**	0.072*
Coparenting cooperation				
Intercept	4.306***^a^	4.096***	4.011***^b^	4.039***^b^
Linear slope	−0.022*	−0.038*	−0.026	0.000
Coparenting conflict				
Intercept	1.734***^a^	1.961***^b^	1.958***	2.041***^b^
Linear slope	0.024***	0.034	0.022	0.001

*Note*. Both secure = children’s attachment security to both mother and father high with security decreasing for both over time; Father secure = children’s attachment security to father higher than security to mother with both decreasing over time; Both insecure = children’s attachment security to both mother and father low with no change over time; Mother secure = children’s attachment security to mother higher than security to father with both increasing over time. All multi-group latent growth curve models controlled for children’s anger/frustration (negative reactivity) temperament. Different superscripts for intercepts across classes indicate significant differences based on pairwise Wald tests. All significant within-class, mother-father differences are reported in the text.

* *p* < .05.

** *p* < .01.

*** *p* < .001.
