# Supplementary material for: Changes in children’s attachment security to mother and father after the birth of a sibling: Risk and resilience in the family
Source: Dev Psychopathol. Author manuscript; Available in PMC 2024 Feb 1. (PMC9192831; doi:10.1017/S0954579421001310)
Supplement: 1 [file NIHMS1747103-supplement-1.docx]

**Table S1**

*Model Fit Indices for Unconditional Linear Models for Parental Sensitivity, Punitive Discipline, and Coparenting*

Note: Parental sensitivity and coparenting were assessed at prenatal, 4, and 8 months; punitive discipline was assessed at 4, 8, and 12 months.

| **Model Fit Index** | Maternal Sensitivity | Paternal Sensitivity | | Maternal Punitive Discipline | Paternal Punitive  Discipline | Mother Coparenting  Cooperation | Father Coparenting  Cooperation | Mother Coparenting  Conflict | Father Coparenting  Conflict |
| --- | --- | --- | --- | --- | --- | --- | --- | --- | --- |
| Linear Model |  | |  |  |  |  |  |  |  |
| *χ*²(1) | 1.912 | | 0.913 | 4.372 | 1.107 | 0.172 | 1.269 | 4.209 | 2.398 |
| *p* | .167 | | .339 | .037 | .293 | .679 | .260 | .040 | .121 |
| CFI | .983 | | 1.000 | .977 | .999 | 1.000 | .999 | .990 | .994 |
| TLI | .948 | | 1.009 | .931 | .998 | 1.007 | .996 | .969 | .983 |
| RMSEA | .062 | | .000 | .126 | .022 | .000 | .034 | .118 | .078 |

**Table S2**

*Summary of Differences Comparing the Both Secure Attachment Class with Other Attachment Configurations on Intercepts (Pre-Birth) and Slopes (Change) for Child, Parent and Coparenting Variables*

| **Both Insecure vs. Both secure** | **Father Secure**  **Mother Insecure vs. Both Secure** | **Mother Secure**  **Father Insecure vs. Both Secure** | **Both Secure vs. Others** |
| --- | --- | --- | --- |
| **Pre-Birth:** | **Pre-Birth:** | **Pre-Birth:** | **Pre-Birth:** |
| **Child:** |  |  |  |
| More externalizing behaviors | More externalizing behaviors | More externalizing behaviors | Less externalizing behaviors |
| More internalizing behaviors | More internalizing behaviors |  |  |
|  |  |  |  |
| **Mother:** |  |  |  |
| Less maternal efficacy | Less maternal efficacy |  |  |
| More maternal parenting stress | More maternal parenting stress | More maternal parenting stress | Less maternal parenting stress |
| Less maternal sensitivity |  |  |  |
| **Father:** |  |  |  |
| Less paternal efficacy | Less paternal efficacy | Less paternal efficacy | More paternal efficacy |
| More paternal parenting stress | More paternal parenting stress | More paternal parenting stress | Less paternal parenting stress |
| More punitive paternal discipline | More punitive paternal discipline |  |  |
| **Coparenting:** |  |  |  |
| Less coparenting cooperation |  | Less coparenting cooperation |  |
| More coparenting conflict | More coparenting conflict | More coparenting conflict |  |
| **Change:** | **Change:** | **Change:** | **Change:** |
| None | Increase in externalizing behaviors | Decrease in externalizing behaviors | Increase in maternal sensitivity |
|  | Decrease in paternal efficacy | Decrease in paternal parenting stress | Increase in paternal sensitivity |
|  | Increase in paternal parenting stress |  | Increase in coparenting conflict |

| **Father Secure/Mother Insecure** | **Mother Secure/Father Insecure** |
| --- | --- |
| **Pre-Birth** | **Pre-Birth** |
| No differences | No differences |
| **Change:** | **Change:** |
| Increase in externalizing behaviors | Decrease in externalizing behaviors |
| No change in maternal efficacy | Increase in maternal efficacy |
| Increase in maternal parenting stress | No increase in maternal parenting stress |
| Increase in paternal parenting stress | No increase in paternal parenting stress |
| Decrease in paternal efficacy | No decrease in paternal efficacy |
| Decrease in coparenting cooperation | No decrease in coparenting cooperation |

**Table S3**

*Summary of Differences Comparing Attachment Configurations of Father Secure/Mother Insecure with Mother Secure/Father Insecure on Intercepts and Slopes for Child, Parent, and Coparenting Variables*

**Figure S1**

*Estimated Mean Trajectories of Maternal and Paternal Self-Efficacy at Prenatal, 1, 4, 8 and 12 Months for Each Family Attachment Class*

**Figure S2**

*Estimated Mean Trajectories of Maternal and Paternal Sensitivity at Prenatal, 4, and 8 months for Each Family Attachment Class*

**Figure S3**

*Estimated Mean Trajectories of Maternal and Paternal Punitive Discipline at 4, 8, and 12 Months for Each Family Attachment Class*

**Figure S4**

*Estimated Mean Trajectories of Mothers’ and Fathers’ Reports of Coparenting Cooperation at Prenatal, 4, and 8 months for Each Family Attachment Class*

**Figure S5**

*Estimated Mean Trajectories of Mothers’ and Fathers’ Coparenting Conflict at Prenatal, 4, and 8 Months for Each Family Attachment Class*
